# Extraskeletal chondroma of the tongue in a two-year-old quarter horse colt: a case report

**DOI:** 10.1007/s11259-025-10918-2

**Published:** 2025-09-29

**Authors:** Maria Virginia Ralletti, Federica Meistro, Maria Adele Tarasconi, Luciana Mandrioli, Andrea Renzi, Riccardo Rinnovati, Alessandro Spadari

**Affiliations:** Department of Veterinary Medical Sciences, University of Bologna, Via Tolara di Sopra 50, Ozzano dell’Emilia, 40064 Italy

**Keywords:** Equine, Horse, Lingual mass, Tongue neoplasm, Chondroma, Extraskeletal

## Abstract

Extraskeletal chondromas (ESCs) are rare benign tumours composed of mature cartilage that typically develop in soft tissues. Their occurrence in horses is extremely uncommon and, to the authors’ knowledge, no previous cases of lingual ESCs in equines have been reported. This report describes a two-year-old Quarter Horse colt presented with progressive dysphagia. Clinical examination revealed a solitary, multilobulated, firm-elastic mass located at the dorsal aboral portion (base) of the tongue. Diagnostic imaging ruled out bone involvement. The mass was surgically excised under general anaesthesia, with a tracheotomy performed to secure the airways; a histopathological diagnosis of ESC was achieved. Postoperative recovery was uneventful, and at six months follow-up, the horse had fully returned to normal feeding behaviour, without signs of recurrence. This appears to be the first described case of an ESC affecting the tongue in horses, adding to the list of differential diagnoses for equine oral masses.

## Background

Extraskeletal Chondromas (ESCs) are benign, cartilaginous tumors that typically develop in soft tissues, without involving adjacent bone or periosteum. These slow-growing, well-demarcated lesions consist of mature hyaline cartilage (Knottenbelt et al. 2015). In humans, ESCs most commonly affect the extremities, particularly the hands and feet (Papagelopoulos et al. 2007; Nazarova et al. 2020), but are also reported in larynx (Lewis et al. 1997), skin (Ando et al. 1995), Fallopian tubes (Han et al. 2002), and very rarely within oral cavity (Vescovi et al. 2014; Attakil et al. 2014).

Reports of ESCs in veterinary literature are exceedingly rare, and the biological behaviour of this type of tumor remains largely undefined. In equine medicine, skeletal chondromas have been documented in anatomic locations such as the paranasal sinuses, nasal cavities, and distal limb bones, including the first phalanx (Pasolini et al. 2009; Seghrouchni et al. 2019). Extra-skeletal variants are extremely uncommon, with isolated cases involving the external ear and larynx (Haridy et al. 2018; Trotter et al. 1981). No previous reports have documented ESCs affecting the equine tongue.

In human literature, lingual ESCs are similarly rare, with only 33 cases reported to date (Attakkil et al. 2014). Histopathologically, these neoplasms consist of lobules of well-differentiated hyaline cartilage and may contain areas of dystrophic calcification or ossification. The etiopathogenesis remains unclear; however, two principal theories are proposed. One suggests that cartilaginous metaplasia results from chronic trauma or irritation with subsequent neoplastic transformation, while the other implicates embryonic remnants of branchial arch cartilage that persist as heterotopic tissue and can proliferate into an appreciable mass (Dimitrijevic et al. 2019).

This report details the clinical presentation, surgical management, cytological and histopathological findings of an ESC located at the dorsal aboral portion of the tongue in a two-year-old Quarter Horse colt. To the authors’ knowledge, this is the first documented case for this location in horses.

## Case presentation

**Fig. 1:**
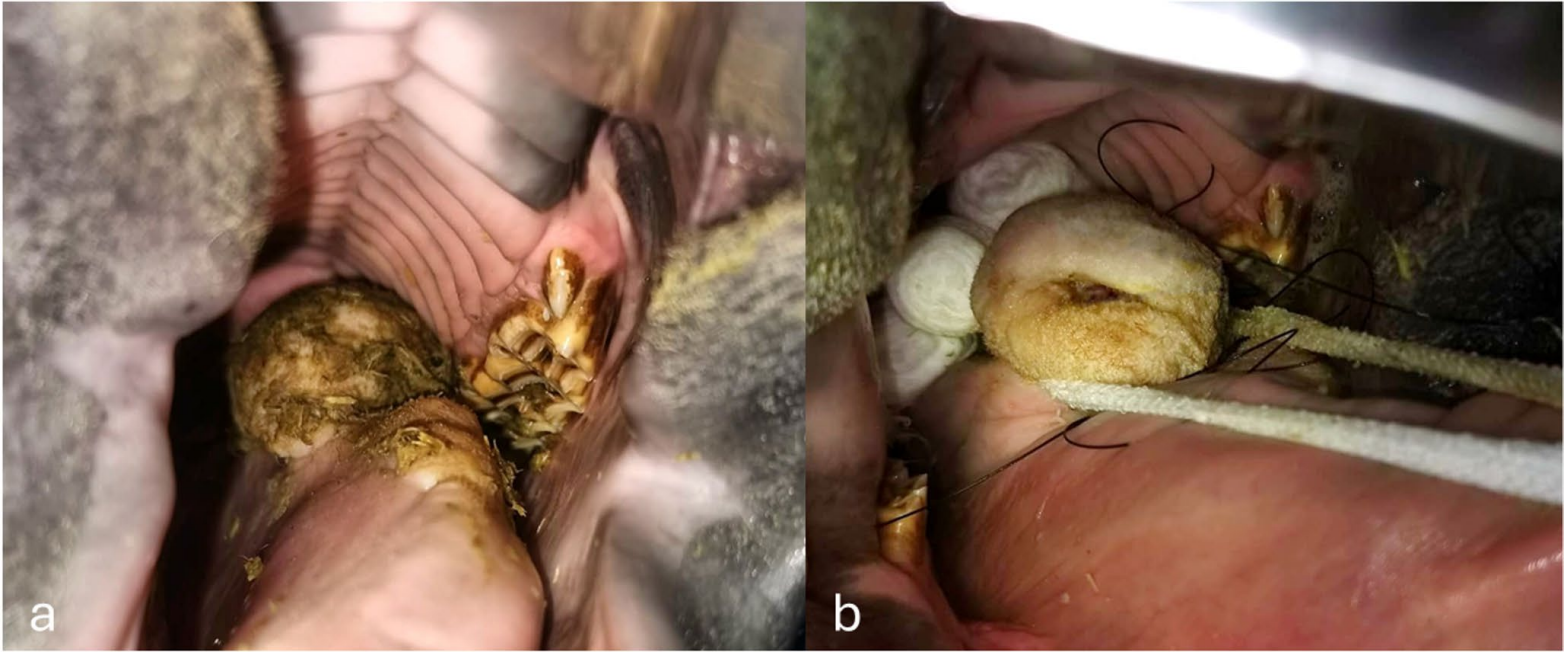
dorsal aspect of the tongue shows the mass in its aboral portion upon oral inspection (**a**) and during surgery (**b**)

A two-year-old Quarter Horse colt was referred to the Veterinary Teaching Hospital presenting progressive dysphagia and suspected presence of an oral mass. Clinical examination revealed a single, multilobulated, whitish mass, approximately 6 cm in diameter, with a firm-elastic consistency, located dorsally on the aboral portion of the tongue (Fig. 1a). Radiographic imaging showed no evidence of bone involvement or mineralization. Considering the horse’s age and the progressive dysphagia and discomfort, surgical excision was recommended.

The procedure was performed under general anaesthesia with the horse positioned in left lateral recumbency. Due to the location of the mass, and the risk of airways compromise, a tracheotomy was performed in advance, to allow safe intubation and secure the airways throughout the surgery. The horse was sedated with romifidine hydrochloride (90 µg/kg). General anaesthesia was induced with ketamine (2.5 mg/kg) and diazepam (0.05 mg/kg) and maintained with isoflurane (1.4%). Intra-operative analgesia included intravenous flunixin meglumine (1.1 mg/kg).

The mouth was held open using a Gunther-type speculum, allowing adequate access to the surgical site. To minimize the risk of blood aspiration, a soft bandaged gauze plug was carefully inserted into the pharynx. The tongue was exteriorized and stabilized using a hemmed gauze bandage around the mass (Fig. 1b). The lesion was excised en bloc with careful dissection, and haemostasis was achieved with electrocauterization. Intraoperative bleeding was minimal. The surgical site was closed with multifilament absorbable sutures in an interrupted pattern. The chosen suture pattern ensured effective alignment and apposition of the tissue margins. The excised mass was submitted to the Pathology Service.

Postoperative management included anti-inflammatory therapy (flunixin meglumine, 1.1 mg/kg IV, SID for four days) and broad-spectrum antibiotics to prevent secondary infection due to the high risk posed by the oral cavity’s constant exposure to feed material and bacterial flora. The selected suture technique following mass excision proved effective, as no wound dehiscence or signs of infection were observed during the immediate postoperative period. Furthermore, tongue mobility appeared to be preserved, suggesting that neither the mass nor the surgical procedure caused significant damage to its neuromuscular components. Tracheotomy wound management included daily dressing changes to allow healing by second intention. Feeding regimen consisted of pelleted hay mash to minimize trauma to the surgical site and ensure suture integrity. The horse was discharged after four days of hospitalization, with instructions for continued wound management. At six months post-surgery, the horse showed no clinical signs of recurrence and had returned to normal feeding behaviour and physical activity. No complications were reported by the owner.

**Fig. 2:**
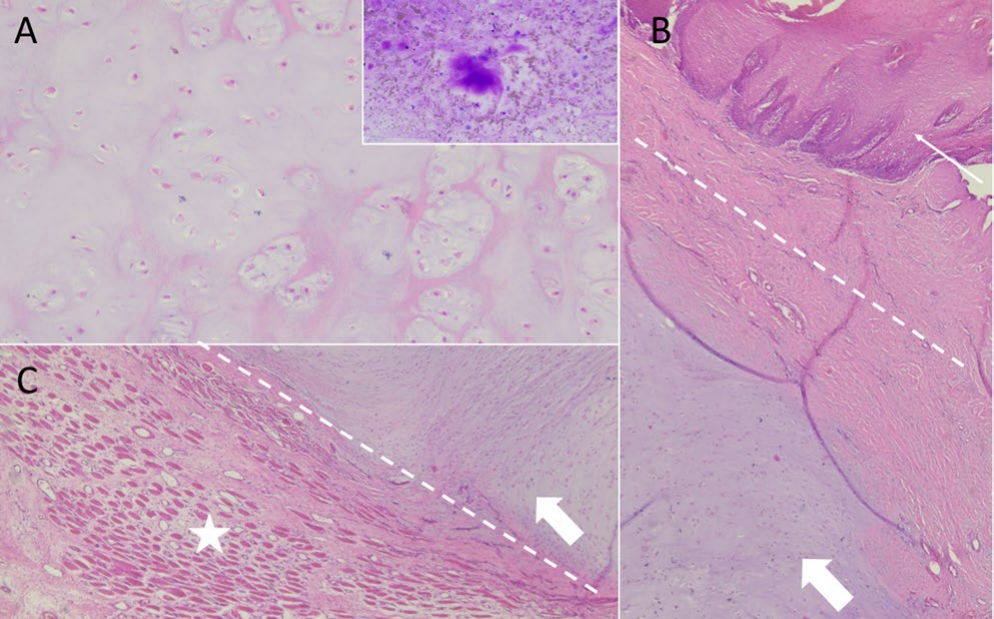
ESC of the tongue, histology and cytology. **A** The neoplastic tissue is characterized by chondrocytes arranged in isogenous groups embedded in an abundant lightly basophilic chondroid matrix (mature hyaline cartilage). FNA cytology (inset) shows absence of criteria of malignancy of the chondrocytes, present as single cells. The deep purple material in the center of the image is the extracellular matrix, MGG staining; magnification is 10X. **B** The mass located in the tongue submucosa (thick white arrow) (mucosal epithelium is pointed out by the thin white arrow), is surrounded by a band of connective tissue (dashes); magnification is 4X. **C** The neoplasm (thick white arrow) exerts compression atrophy of the glossal myofibres (white star). Hematoxylin and eosin staining; magnification is 4X

Following surgical excision, fine-needle aspiration (FNA) sampling from the mass was carried out. After drying, the cytological slides were stained using May-Grünwald Giemsa (MGG), mounted with cover slips and analyzed under an optical microscope (Nikon, Eclipse E600). Cytology showed a population composed of round to ovoid cells in a background of extracellular pink material (Fig. 2A inset), with a lacking of nuclear and cytoplasmic criteria of malignancy. A suspicion of a neoplastic process was made.

Samples from surgical excised mass were subjected to standard procedures for histological evaluation (fixation in buffered formalin, paraffin embedding, and Hematoxylin-Eosin staining). Histological examination confirmed the cytological findings of a neoplasia. A multilobulated, well-demarcated, submucosal mass was composed of well-differentiated chondrocytes embedded in abundant extracellular chondroid matrix (Fig. 2A). The neoplastic tissue was surrounded by a dense band of mature connective tissue (Fig. 2B dashes). Chondrocytes were small, with low amount of cytoplasm and small intensely basophilic nucleus; in some areas they replicated the appearance of hyaline cartilage, as they were arranged in isogenous groups. No mitotic figures were found. There was lack of infiltrative pattern. The submucosal mass exerted compression atrophy of the adjacent skeletal myofibers; oedema and proliferation of endomysial connective tissue were detected as well (Fig. 2C).

The clinico-pathological findings were indicative of a lingual ESC.

## Discussion and conclusion

In equids, chondromas more frequently affect skeletal structures, while the few reports of extra-skeletal variants suggest a predilection for isolated anatomical locations (external ear, skin and larynx), without clear etiological patterns. According to Knottenbelt’s Equine Oncology, benign tumors of cartilaginous origin, including chondromas, are infrequent findings in equine practice. Their biological behavior, clinical progression, and recurrence rates remain largely undefined (Knottenbelt et al. 2015). Berrocal et al., reviewing cartilaginous and non-cartilaginous tracheal tumors in dogs and cats, state that incidence in young animals and rarity suggest that a chondroma could be a dysplastic lesion of proliferative disorganized cartilage without central core ossification, differentiating it from osteochondroma (Berrocal et al. 2025; Caswell and Williams 2016).

In the present case, the absence of reported trauma and anatomical abnormalities that could cause repeated tissue damage, associated to the histological appearance, favour an embryonic etiopathogenesis. However, the equine tongue is constantly exposed to mechanical stress due to its functional involvement in prehension, mastication, and deglutition, suggesting a predisposition to chronic microtrauma. For that reason, traumatic etiology should not be excluded.

When evaluating equine oral masses, a comprehensive list of differential diagnoses should be considered, with both benign and malignant processes. Several benign conditions may present similarly, among them papillomas, fibromas, lipomas, and non-neoplastic conditions as granulomas. On the other hand, malignant lesions that need to be ruled out include squamous cell carcinoma, melanoma, and chondrosarcoma (Pasolini et al. 2009; Seghrouchni et al. 2019).

Cytology is a rapid and cost-effective method able to differentiate between inflammatory reaction and neoplasia. FNA sampling was suggestive of a neoplasm. The postoperative histological samples provided complementary information regarding the biologic growth pattern of the mass, which was not infiltrative but showed a mass effect causing compression atrophy of the tongue myofibers.

A cartilage tumor is considered malignant (chondrosarcoma) when the following histological features are found: many cells with plump nuclei, more than an occasional cell with two nuclei, and especially giant chondrocytes with single or multiple nuclei, clumps of chromatin, prominent nucleoli and irregularly shaped nuclei. The absence of mitotic figures does not rule out malignancy, since cell division in chondrosarcomas may be amitotic (Ramìrez et al. 2015). In humans, low grade chondrosarcoma can be difficult to differentiate from chondroma; a combination of clinical presentation, radiologic appearance, and cytologic interpretation is needed, and biopsy is typically required for a definitive diagnosis (Ramìrez et al. 2015; Lawrence and Milne 2021). Chondromas, the benign counterpart of chondrosarcomas, are rare tumors appearing as well circumscribed, dense lesions that are rarely painful on palpation. Aspiration of benign lesions can be unrewarding due to low cellularity, although if the sample is adequate, cytology can guide further diagnostics (Lawrence and Milne 2021); absence of nuclear atypia, necrosis, and invasive character are histological features indicative of a benign tumor (Ramìrez et al. 2015).

According to the authors’ knowledge, one description of chondrosarcoma of the tongue in a young horse is available; unfortunately, any microscopic image contribution in the paper is available, but histopatological description reports intense cellular infiltrate composed by irregular densely arranged spindle shaped cells with indistinct borders; nuclei were deeply basophilic with a stippled chromatin pattern and average mitoses count was two per 400X field. The cells were also producing a lightly basophilic staining chondroid matrix. Eleven months after surgery, the horse has maintained normal body weight and was asymptomatic, with no evidence of local recurrence or distant metastasis on clinical examination (Wilson and Anthony 2007).

In the current report, the identification of well-differentiated chondrocytes embedded within an abundant hyaline matrix, absence of mitoses or atypical features, and lack of invasive growth were consistent with a diagnosis of ESC. In human medicine, a case report about lingual chrondroma was described as a mass covered with normal tongue mucosa and histologically as multilobulated areas composed by mature hyaline cartilage, surrounded by a capsule of fibrous tissue, and abundant extracellular matrix in which cartilaginous cells are embedded (Park et al. 2022); these findings are consistent with those from our case.

En bloc resection was facilitated by tracheotomy, which reduced the risk of airway obstruction and aspiration. The location of the mass raised concerns about possible intraoperative complications, including haemorrhage or oedema. Since the surgical site was constantly exposed to feed material and bacterial contamination, antibiotic treatment was administered to reduce the risk of secondary infections and delayed wound healing. Postoperatively, care was aimed at reducing trauma to the surgical site and maintaining adequate feeding to ensure optimal healing.

Preservation of tongue mobility indicated that the dysphagia was likely due to mechanical interference, rather than any true functional impairment.

No recurrence was reported at six months post-surgery, supporting a favourable prognosis. However, continued follow-up is advised.

To the best of the author’s knowledge, this case report documents the first occurrence of a lingual ESC in a horse.

Given the rarity of this condition, and its potential to mimic more aggressive diseases, such as chondrosarcoma, accurate histopathological diagnosis is essential for optimal case management. Early identification and surgical intervention are deemed necessary to avoid functional consequences.

Furthermore, this case also underlines the possibility of considering ESCs in the differential diagnosis when evaluating equine oral masses. Additional cases are necessary to contribute to a more accurate understanding of the biological behaviour, optimal management strategies, and long-term outcomes of ESCs in horses.

## Data Availability

No datasets were generated or analysed during the current study.
